# Editorial for the Special Issue “Early Molecular Diagnosis and Comprehensive Treatment of Tumors”

**DOI:** 10.3390/cimb47110954

**Published:** 2025-11-17

**Authors:** Yao-Chou Tsai, Chan-Yen Kuo

**Affiliations:** 1 Division of Urology, Department of Surgery, Taipei Tzuchi Hospital, The Buddhist Tzu Chi Medical Foundation, New Taipei City 231, Taiwan; tsai1970523@yahoo.com.tw; 2 College of Medicine, Buddhist Tzu Chi University, Hualien 970, Taiwan; 3 Institute of Oral Medicine and Materials, College of Medicine, Tzu Chi University, Hualien 970, Taiwan

Cancer is one of the leading causes of morbidity and mortality worldwide, and it poses a persistent challenge to modern medicine [1]. Despite major advances in cancer biology, diagnostics, and therapeutics, the complexity and heterogeneity of tumors continue to hinder early detection and effective treatment [2]. In recent years, however, transformative progress in molecular biology has led to the emergence of powerful tools that enable relatively early diagnosis, personalized therapy, and improved clinical outcomes [3,4]. In this context, we present this Special Issue of Current Issues in Molecular Biology, titled “Early Molecular Diagnosis and Comprehensive Treatment of Tumors.” This Special Issue presents five high-quality articles, including three original research papers, one review article, and one case report, that collectively highlight emerging concepts and technological advances in tumor detection and treatment. The contributions of these studies reflect interdisciplinary efforts that bridge basic science, translational research, and clinical applications.

Sarf and Bel’skaya [5] investigated alterations in nitrogen metabolism in breast cancer by analyzing levels of urea, branched-chain amino acids (BCAAs), glutamine (Gln), and glutamate (Glu) in saliva. Saliva samples from patients with breast cancer, fibroadenomas, and healthy controls demonstrated that salivary urea levels progressively increased from healthy individuals to benign breast disease and were highest in breast cancer patients, particularly in luminal A and luminal B subtypes. This elevation may reflect reduced urea utilization owing to altered oral microbiota diversity and estrogen-dependent effects common in luminal tumors. Amino acid analysis revealed increased levels of BCAAs and Glu in breast cancer saliva, while Gln was decreased in most subtypes except in relatively aggressive phenotypes such as luminal B HER2-positive tumors and triple-negative breast cancer (TNBC), which showed markedly elevated Gln levels and a high Gln/Glu ratio. These patterns indicate enhanced nitrogen redistribution and metabolic reprogramming in aggressive tumors to support rapid proliferation and biosynthesis. Overall, the findings suggest that salivary nitrogen metabolites reflect tumor metabolic activity and phenotype-specific alterations; urea levels may be useful as a non-invasive indicator of microbiome-related metabolic dysregulation in breast cancer, while amino acid profiling, particularly the Gln/Glu ratio and BCAA levels, may provide insight into tumor aggressiveness and catabolic activity, thereby offering a potential tool for metabolic monitoring and early detection in breast cancer [5].

To explore their potential role as biomarkers reflecting tumor biology and immune modulation, Kabut et al. [6] investigated the serum concentrations of interleukin-21 (IL-21) and interleukin-22 (IL-22) in women with invasive breast cancer compared to those with benign breast lesions. Although both cytokines participate in tumor–immune interactions by exerting either pro- or anti-tumor effects depending on the microenvironment, the findings of this study did not reveal statistically significant differences in IL-21 or IL-22 serum levels between malignant and benign groups. Similarly, no associations were found between cytokine levels and tumor grade or molecular subtype, suggesting that systemic levels of these interleukins may not directly mirror tumor aggressiveness or molecular characteristics at this stage of investigation. Notably, a significant negative correlation was observed between IL-21 and IL-22 concentrations exclusively in the benign lesion group, implying that regulatory cytokine balance may be preserved in non-malignant conditions but is disrupted in invasive cancer, possibly owing to tumor-induced immune dysregulation. These findings highlight complex cytokine interactions within the tumor microenvironment and support the hypothesis that serum cytokine alterations may reflect early immune changes even in benign lesions. Nevertheless, the limited sample size, particularly within molecular subgroups, restricts the statistical power and generalizability of these observations. Future large-scale and multicenter studies integrating serum, tissue-level expression analyses, and functional immune assays are essential to clarify the pathophysiological relevance of IL-21 and IL-22 in breast cancer and evaluate their potential utility as prognostic or immunotherapeutic biomarkers. Overall, the present results provide a foundation for further investigation into cytokine-mediated mechanisms in breast cancer pathogenesis and highlight the need for deeper immunological profiling to advance precision oncology [6].

Kan et al. [7] validated the prognostic value of three circulating cell-free RNA (cfRNA) biomarkers including HPGD, PACS1, and TDP2 in colorectal cancer (CRC) using TaqMan qPCR and correlation analysis in a cohort of 52 patients. Consistent with previous RNA sequencing data, HPGD was significantly upregulated post-operation and demonstrated a strong association with patient survival, where lower HPGD expression was correlated with poorer outcomes, supporting its role as a protective tumor suppressor gene and reliable negative prognostic indicator. PACS1 showed significant downregulation following surgery and revealed notable correlations with both HPGD expression and survival time, indicating its potential contribution to tumor progression dynamics and its relevance in postoperative prognostic evaluation. TDP2 expression displayed variability; although not significantly altered overall post-operation, its relatively high expression was strongly correlated with recurrence risk, suggesting a mechanistic involvement in DNA repair pathways that may contribute to therapeutic resistance and tumor relapse. Collectively, these findings demonstrate that cfRNA biomarkers carry meaningful prognostic information reflective of tumor burden and disease trajectory. Despite limitations such as relatively small sample size and single-center design, this study supports the integration of minimally invasive cfRNA profiling into CRC management to aid early risk stratification, recurrence monitoring, and personalized treatment planning. Future multicenter investigations with larger and more diverse populations, along with longitudinal sampling, are warranted to validate these biomarkers and enhance their clinical utility in precision oncology [7].

The review [4] conducted by Hsu et al. highlights the importance of integrating early molecular diagnostics with comprehensive and personalized treatment strategies to improve outcomes in oral squamous cell carcinoma (OSCC). Advances in next-generation sequencing, salivary liquid biopsy, and exosomal microRNA profiling such as miR-1307-5p and miR-21 provide promising non-invasive tools for early detection and disease monitoring. Molecular alterations including TP53 mutation, CDKN2A inactivation, and EGFR overexpression contribute to OSCC pathogenesis and offer opportunities for biomarker-based risk stratification. Innovative imaging technologies such as fluorescence molecular imaging and structured illumination fluorescence lifetime imaging microscopy enhance early lesion detection and intraoperative precision. Therapeutically, multimodal regimens combining surgery, concurrent chemoradiotherapy, neoadjuvant chemotherapy, metronomic chemotherapy, immunotherapy, and molecularly guided precision medicine are redefining OSCC management. Despite these advances, challenges persist in biomarker standardization, tumor heterogeneity, and translating molecular findings into routine clinical practice. Future efforts must emphasize multicenter validation of salivary and circulating biomarkers, AI-driven diagnostic integration, and development of personalized therapeutic algorithms that improve survival while preserving quality of life. Ultimately, combining molecular diagnostics with precision oncology offers a transformative framework for earlier intervention, treatment optimization, and long-term disease control in OSCC [8].

The case report by Albu et al. [9] presents a three-year-old child with Down syndrome who developed B-cell acute lymphoblastic leukemia (DS-ALL) characterized by five concurrent high-risk genomic lesions including NRAS p.G13D, JAK2 p.R683G, EP300 p.Q1766*, P2RY8-CRLF2 fusion, and iAMP21, an exceptionally rare molecular profile not previously reported in combination. Despite this adverse genomic background traditionally associated with treatment resistance and poor outcomes, the patient achieved complete remission with undetectable minimal residual disease by Day 33 following risk-adapted induction chemotherapy. The clinical course was complicated by severe infectious events and chemotherapy-related dental pathology, both effectively managed through proactive multidisciplinary supportive care that included infectious disease monitoring, cardiology-guided chemotherapy adjustments for congenital heart disease, and early dental intervention to prevent oral infection. This case highlights the critical value of comprehensive molecular diagnostics for precise risk stratification and therapeutic planning in DS-ALL, and highlights that precision supportive care is as important as cytotoxic therapy in determining outcomes. Although limited by short-term follow-up and its single-patient design, this report provides clinically meaningful insights into the integration of genomic profiling, MRD surveillance, and coordinated multisystem supportive strategies to optimize treatment tolerance and survival in this high-risk population. Future multicenter studies are warranted to evaluate the effect of complex genomic interactions on treatment response and to develop DS-specific therapeutic algorithms that improve long-term prognosis and quality of life [9].

## Data Availability

Not applicable.

## Abbreviations

The following abbreviations are used in this manuscript:

BCAAs	Branched-chain amino acids
cfRNA	Circulating cell-free RNA
CRC	Colorectal cancer
DS-ALL	Developed B-cell acute lymphoblastic leukemia
Gln	Glutamine
Glu	Glutamate
OSCC	Oral squamous cell carcinoma
TNBC	Triple-negative breast cancer
